# Heparan sulfate is the attachment factor associated with channel catfish virus infection on host cells

**DOI:** 10.3389/fvets.2023.1260002

**Published:** 2023-09-07

**Authors:** Fei Yu, Hongxun Chen, Jiehua Xu, Yu Wang, Chunlan Nie, Siyang Song, Lihui Meng, Kai Hao, Zhe Zhao

**Affiliations:** Jiangsu Province Engineering Research Center for Marine Bio-resources Sustainable Utilization, College of Oceanography, Hohai University, Nanjing, China

**Keywords:** channel catfish virus, heparin, attachment factor, virus infection, antiviral agents

## Abstract

Channel catfish virus (CCV; family *Alloherpesviridae*) infects channel catfish, causing great harm to aquaculture fisheries and economic development. Attachment is the first step in viral infection and relies on the interaction of virions with components of the extracellular matrix (ECM). The present study aimed to explored the role of the main three ECM components in CCV attachment. Western blotting and quantitative real-time PCR analysis showed that neither collagen nor hyaluronic acid treatments had significant effects on CCV attachment. When exogenous heparin was used as a competitive inhibitor, the adhesion of heparin sodium salt to CCV was dose-dependent. When the concentration of heparin sodium salt was 10 mg/mL, the inhibitory effect on CCV infection of channel catfish ovary (CCO/BB) cells was more than 90%. Heparinase I could significantly prevent CCV attachment by digesting heparan sulfate on the cell surface, and both heparin sodium salt and heparinase I could dose-dependently reduce CCV titers, suggesting that heparin plays an important role in CCV attachment. In addition, the binding experiments between heparin-agarose beads and virions showed that CCV virions could specifically bind to heparin in a dose-dependent manner. The above results suggested that heparan sulfate might be an attachment factor involved in CCV infection of CCO/BB cells. These results increase our understand of the attachment mechanism of CCV and lay the foundation for further research on antiviral drugs.

## Introduction

Channel Catfish Virus (CCV) is an enveloped double-stranded DNA virus, also known as Ictalurid herpesvirus I, which is an important member of the virus family Alloherpesviridae (1). The virion is about 175–200 nm in size and consists of an envelope, an interlayer, and a nucleocapsid. The nucleocapsid has an icosahedral structure and consists of 162 capsomers with a diameter of about 100 nm (2). The genome size of CCV is ~134 kb, and the entire genome is predicted to consist of 76 open reading frames (ORFs). With the continuous expansion of channel catfish breeding, channel catfish diseases are becoming increasingly serious, the most harmful of which is channel catfish virus disease (CCVD) caused by CCV (3, 4). CCV outbreaks in channel catfish aquaculture have caused serious economic losses.

Virus infection is a complex process that begins with the interaction of virus-adsorbing proteins (VAPs) with receptors on the surface of target cells. The binding between the virus and the target is relatively unstable and vulnerable to external physical and chemical factors; however, it can provide an attachment site for the virus, such that the virus can be trapped on the surface of the cell from the external environment. The adsorption of viruses has a certain selectivity. The adsorption of some viruses requires the identification of specific modification patterns or groups of surface molecules on target cells. The infectivity and tropism of viruses to cells or tissues are affected by adsorption (5). Virus adsorption and entry can be mediated by one or more adsorption receptors. Adsorption receptors can mediate virus retention on the cell surface and promote virus infection, but cannot mediate virus entry into cells, and are usually cell surface polysaccharides, glycoproteins, and glycolipids. Viruses can greatly increase the strength of the interaction through multivalent binding to cell surface adsorbed receptors (6).

The extracellular matrix (ECM) is an acellular macromolecular network composed of various glycoproteins (7). It participates in physiological functions, such as signal transduction, cell recognition, cell migration, and differentiation (8, 9). Sialic acid (SA) and glycosaminoglycans (GAGs) are the main adsorption factors identified to date. The process of virus adsorption by recognizing sialic acid on the cell surface is a classical virus-host interaction, and there are many modifications and variants of sialic acid (10). Many viruses, such as influenza A virus (11) and avian infectious bronchitis virus (12), use sialic acid as an adsorption receptor. GAGs play an important role in viral attachment and are capable of specific recognition and signaling (13), usually serving as viral attachment factors in mammals (14). GAGs are mainly divided into four categories: hyaluronic acid without charge, keratan sulfate (KS) with the shortest skeleton, chondroitin sulfate (CS) with more negative charges, and heparan sulfate (HS) with the most negative charges. HS is one of the most common GAGs in mammals and is involved in the cell attachment of many viruses, such as herpes simplex virus (HSV) and respiratory syncytial virus. Cell surface GAGs are composed of complex linear polysaccharides and are ubiquitously expressed in most cell types (15). GAGs on the cell surface can be covalently linked to proteins in the form of macromolecular side chains to form proteoglycans (PGs), thereby regulating the activity of extracellular factors and participating in signal transmission.

Research on adsorption factors is of great theoretical significance to determine the pathogenic mechanism of channel catfish herpes virus. Previous studies have shown that collagen (16), hyaluronic acid (17), and HS (18) interfere with HSV-1 activity, and hyaluronic acid usually exists in the free form or is non-covalently bound to other molecular surfaces to facilitate viral adsorption (19). Collagen also has the function of binding various basement membrane and ECM proteins (20). HS has the strongest ability to mediate adsorption among GAGs side chain members, and is often an important object of virus adsorption research. Heparan (HP) in the cell membrane and cell matrix is composed of repeating disaccharide units (D-glucose). As a highly sulfated polysaccharide chain (composed of uronic acid and D-N-acetylglucosamine), exogenous heparan molecules and analogs can adjust the affinity of HS and viral ligands, and can be used to control a variety of virus infections, with potential antiviral value (21). Although the research methods for viral adsorption factors are relatively complete, so far no research results from the adsorption molecules applied to fish herpes virus pathogen control have been published. This paper adopted CCV as the main research object. CCV and HSV-1 belong to the family Herpesviridae. The three adsorption factors, hyaluronic acid, collagen, and heparan were selected to identify the adsorption factors related to CCV infection, which will provide a basis for determining the pathogenic mechanism of CCV and is of great theoretical significance to provide targets for the development of antiviral drugs.

## Materials and methods

### Cells and viruses

The channel catfish ovary (CCO) cell line is a brown bovine head cell line known as CCO/BB (22). Cells used in this study were maintained in Dulbecco's modified Eagle medium (DMEM) containing 10% fetal bovine serum (Thermo Fisher, Waltham, MA, USA), penicillin (100 U/mL), and streptomycin (100 μg/mL) at 28°C. The CCV strain VR-665 (kindly provided by Prof. Jun-Fa Yuan, Huazhong Agricultural University, China (23, 24), was maintained in our laboratory and used in the present study. CCO cells were used for CCV propagation, and CCV titers were determined using median tissue culture infectious dose (TCID_50_) assays on CCO cells, as previously described (23).

### Reagents

Heparan sodium and collagen were purchased from Sangon Biotech (Shanghai, China). Hyaluronic acid was purchased from Selleck Chemicals (Houston, TX, USA). Heparinase I was purchased from Sigma-Aldrich (St. Louis, MO, USA). All drugs except heparinase I were dissolved in water according to the manufacturer's instructions and then stored as stock solution at −80°C. Heparinase I was re-suspended in phosphate-buffered saline (PBS) at 100 U/mL and stored at −20°C.

### Viral culture

Cells were infected with virus at a multiplicity of infection of 0.01 for 6 days. After three freeze-thaw cycles, the supernatant was collected by centrifugation at 6,000 × g for 40 min at 4°C. The pellet was re-suspended in PBS and stored at −80°C. The intracellular virus growth curve of CCV was determined as previously described (25).

### Virus titration

Viral titers were determined using the TCID_50_ assay. Briefly, CCO/BB cells were seeded in 96-well plates and incubated overnight at 28°C to form a confluent monolayer prior to infection. Ten-fold serial dilutions of virus were added to the wells of the plates and absorbed for 4 h at 28°C. Thereafter, unbound virus was removed and the cells were washed twice with DMEM. The cells were incubated with DMEM at 28°C and the cytopathic effect (CPE) was observed after 2 days. Drug-treated groups were incubated with CCO/BB cells for 1 h before CCV infection, and the TCID_50_ was calculated using Reed and Munch mathematical analysis (26).

### Inhibitor cytotoxicity test

Cytotoxic effects of all drugs were examined using the 3-(4,5-dimethylthiazol-2-yl)-2,5-diphenyltetrazolium bromide (MTT) assay. In brief, CCO/BB cells were seeded in a 96-well plate overnight at 28°C before treatment. Each inhibitor was diluted in different concentration gradients according to its solubility. Then, medium was changed to one containing different concentrations of the inhibitors. After 24 h of incubation, 10 μL of MTT solution was added to each well and incubated for 4 hours. The cell viability analysis was performed using a MTT Cell Proliferation and Cytotoxicity Assay Kit (Sangon Biotech). Optical density was measured at 570 nm using a Spark microplate reader (TECAN, Männedorf, Switzerland). The optical density of the control group was set to 100%. Three parallel wells were used for each experiment.

### Inhibition assay

To analyze CCV uptake, quantitative real-time PCR (qPCR) was used to analyze the gene copy number of the virus. CCO/BB cells were seeded in 24-well plates and incubated overnight at 28°C before infection. Then, CCO/BB cells were pretreated with heparin sodium salt, hyaluronic acid, and collagen for 1 h at 28°C, respectively. CCV was then added to the medium at a multiplicity of infection of 0.01 and incubated at 28°C for 4 h. The inoculum was removed, the cells were washed twice with DMEM medium, the DMEM was replaced and the cells were incubated for about 24 h at 28°C. Cells were harvested by centrifugation for viral DNA extraction and western blotting analysis. The ultrastructure of the virions in infected cells was examined using transmission electron microscopy (TEM) (Axio Vert A1, 10X; Carl Zeiss, Oberkochen, Germany). To confirm the effect of the drug on the CCV infection of CCO/BB, the drug was first incubated with cells or virus (pre or post), respectively.

### Heparinase treatment

To further investigate the role of adsorption factors in virus binding, cells were treated with heparinase I to remove HS from the cell surface. Heparinase I was dissolved in 1 × PBS at 100 U/mL before the assay and stored at −20°C. CCO/BB cells were seeded in 24-well plates and cultured at 28°C overnight, the medium was removed before the assay, cells were incubated with different concentrations of heparinase I for 1 h, and then washed twice with fresh medium. CCV was added to the medium at a multiplicity of infection of 0.01 and incubated for 4 h at 28°C. After 4 h of incubation, the cells were washed twice with fresh medium and then the medium replaced with DMEM medium. After 24 h of culture, the cells were collected for western blotting or qPCR analysis. The ultrastructure of the virions in infected cells was examined using TEM (Axio Vert A1, 10X).

### Heparan–Sepharose binding assays

Heparan-Sepharose beads 6FF (Sangon Biotech) or control Sepharose beads (Sigma) were washed three times with PBS at room temperature and equilibrated, then 100, 300, or 500 μL of CCV was added, and the mixture was shaken at low speed at 4°C for 2 to 3 h. Unbound virions were collected and the pellet was washed three times with PBS. Samples were re-suspended in 5 × protein sample buffer containing 5% 2-mercaptoethanol, subjected to electrophoresis, and detected using western blotting analysis. The eluted CCV virus particles were treated using the negative staining method and observed using TEM.

### DNA extraction

Total DNA was extracted from the collected CCO/BB cells using a viral RNA/DNA extraction kit (Takara, Dalian, China) according to the manufacturer's instructions. Briefly, an equal volume of Buffer VGB was added, and the Carrier RNA and Proteinase K were added to remove excess viral RNA and protein. Carrier RNA and Proteinase K must be stored at −20°C and operated on ice. The mixture was kept at 56°C for 10 min to fully lyse the virus. After adding absolute ethanol, the mixture was centrifuged at 14,000 × g for 2 min. Further processing was carried using Buffer RWA and Buffer RWB. Absolute ethanol should be added to Buffer RWB before first use to ensure elution efficiency. Finally, RNase-free ddH_2_O was added and the viral DNA was eluted by centrifugation. The mixture was allowed to stand at room temperature for 5 minutes before centrifugation to ensure reaction efficiency. All reagents used, except Carrier RNA and proteinase K, were stored at room temperature. The extracted DNA was stored at −20°C for subsequent quantitative PCR analysis.

### Quantitative real-time PCR

qPCR was performed on the LightCycler^®^ 96 System (Roche, Basel, Switzerland). The cycling conditions were: An initial denaturation at 98°C for 60 s, followed by 35 cycles of 98°C for 10 s, 60°C for 15 s, and 72°C for 30 s. The copy number of the viral genomic DNA was determined according to the copy number of the CCV ORF39 gene. A known amount of the pGBKT7-ORF39 plasmid containing the CCV ORF39 gene was serially diluted 10-fold as a standard. The standard curve of the cycle threshold values was determined, a standard curve of CCV ORF39 was established, and the viral gene copy number was calculated according to the standard curve. The primer sequences for CCV ORF39 are as follows, forward: 5′-GAAGATAGCCCGTCTCACCG-3′; reverse: 5′-ATCTCGATCAGCATCTGGCG-3′. The pGBKT7 plasmid is maintained in our laboratory. A ChamQ Universal SYBR qPCR Master Mix was purchased from Vazyme (Nanjing, China) and stored at −20°C. qPCR was performed in triplicate in each sample. All qPCR reactions were performed in triplicate. The resulting data were analyzed using the LightCycler^®^ 96 SW 1.1 software (Roche).

### Western blotting

For western blotting analysis, cells were lysed with cell lysis buffer (Beyotime, Jiangsu, China) and the protein concentration was determined using the Bradford method. Proteins obtained after lysing the cells were separated by 10% sodium dodecyl sulfate-polyacrylamide gel electrophoresis and transferred to polyvinylidene fluoride membranes (Pall, Shanghai, China) using a semi-dry transfer cell (Bio-Rad, Hercules, CA, USA) at 120 V about 20 min. The membrane was then further incubated with anti-ORF39 (prepared in our laboratory) (27) or anti-glyceraldehyde-3-phosphate dehydrogenase (GAPDH) monoclonal antibodies (ABclonal, 1:2,000). Horseradish peroxidase-conjugated goat anti-rabbit IgG or goat anti-mouse IgG (Sigma, 1:4000) were used as secondary antibodies. Immunoreactive protein bands were detected using an ECL kit (Tiangen Biotechnology, Beijing, China).

### Statistical analysis

Statistical analyses were expressed as means ± SD and performed using GraphPad PRISM software version 8 (GraphPad Software, San Diego, CA, USA). The probability of *P* < 0.05 was considered to be statistically significant.

## Results

### Toxicity testing of ECM compounds and enzymes on CCO/BB cells

**Figure 1:**
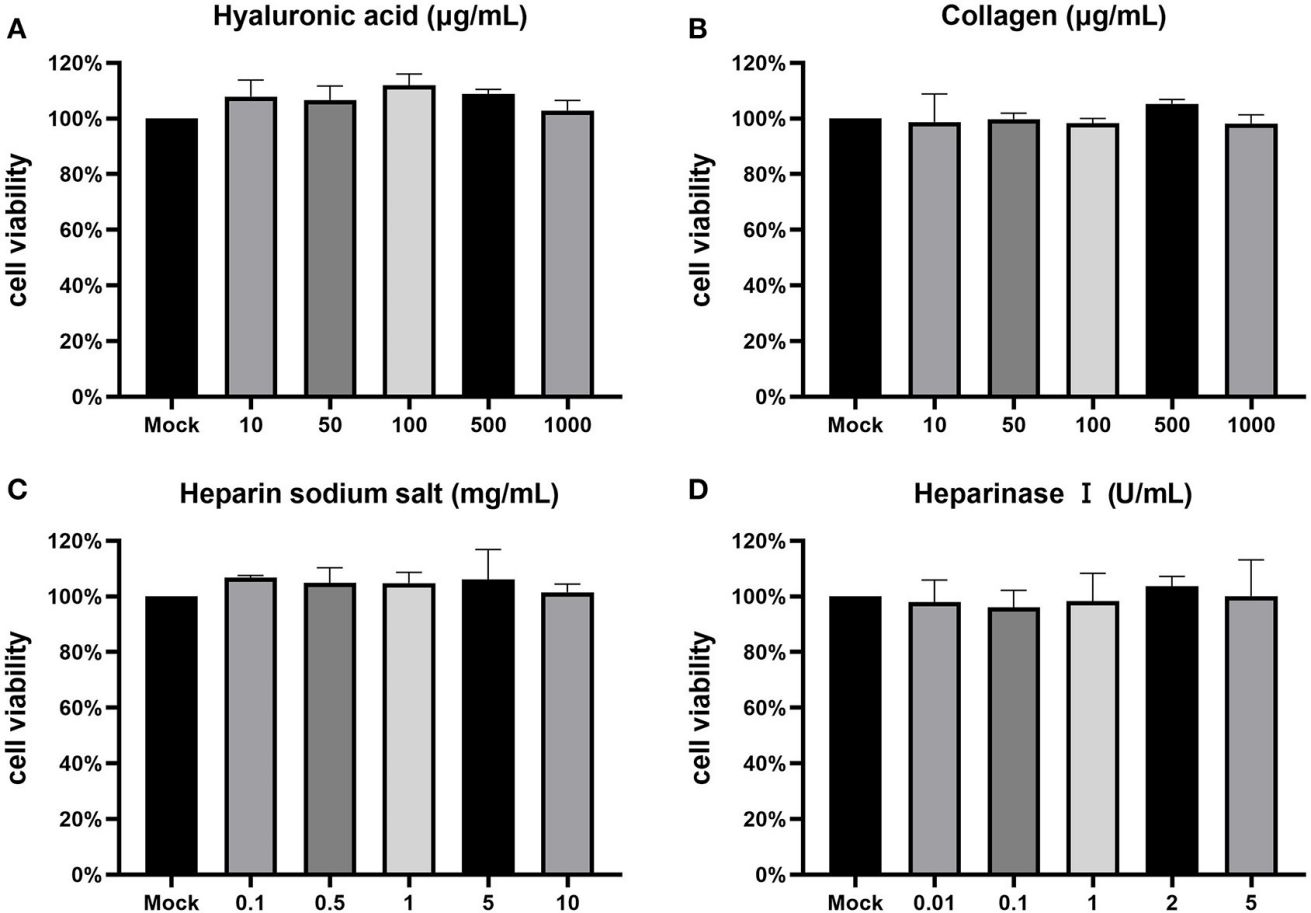
Cytotoxicity test. The toxicity of inhibitors on CCO/BB cells was measured using an MTT assay and calculated as a percentage of viable, non-treated cells. Cells were incubated with different concentrations of hyaluronic acid **(A)**, collagen **(B)**, heparin sodium salt **(C)**, heparinase I **(D)**. Values represent mean ± SD (*n* = 3) for experiments performed in triplicate. CCO/BB, channel catfish ovary cells; MTT, 3-(4,5-dimethylthiazol-2-yl)-2,5-diphenyltetrazolium bromide.

To study the effect of various adsorption factors on CCV infection of CCO/BB cells, their safe concentrations on CCO/BB cells was tested first. To this end, different concentrations of drugs were added to the CCO/BB cell culture supernatant, and cytotoxicity was tested using the MTT assay after 24 h. The MTT method is a common method for activity detection of active factors, drug screening, and cytotoxicity assay. It is widely used for the *in vitro* detection of changes in cell proliferation activity caused by inducers and inhibitors. Succinate dehydrogenase in the mitochondria of living cells can reduce the exogenous MTT solution to blue-violet crystalline formazan and deposit it in the cells, thereby detecting cell viability. In the presence of specific solvents, formazan can be completely dissolved. Figure 1 shows that after the cells were treated with hyaluronic acid at a concentration of <1 mg/mL, there was no significant change in the morphology of the cells under the microscope, and the cell viability was above 95%. After the cells were treated with collagen at a concentration of <1 mg/mL, there was no significant change in the morphology of the cells under the microscope, and the cell viability was above 95%. After the cells were treated with heparin sodium salt at a concentration of <10 mg/mL, the morphology of the cells did not change significantly under the microscope, and the cell viability was above 95%. The cell viability was higher after cells were treated with heparinase I at a concentration of <5 U/mL. The results showed that CCO/BB cells had good tolerance to ECM components, and the cells could be treated with higher concentrations of these drugs without significant cytotoxicity.

### Roles of major adsorption factors in CCV attachment

**Figure 2:**
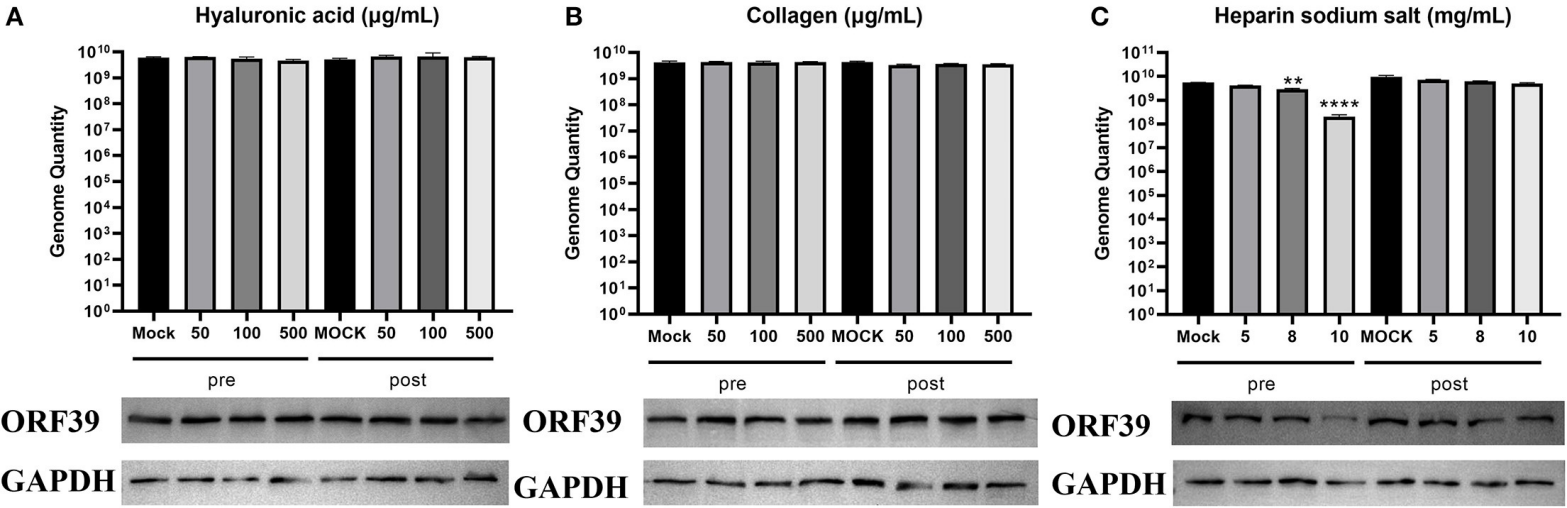
Heparin sodium salt can effectively inhibit CCV attachment. Channel catfish ovary (CCO) cells were treated with hyaluronic acid, collagen, or heparin sodium salt 1 h before (pre-inoculation) or post-inoculation with CCV. **(A)** Analysis of the cells treated with hyaluronic acid. Cells were harvested at 24 h post-infection (hpi), lysed, and analyzed using western blotting or qPCR analysis. **(B)** Analysis of the cells treated with collagen. Cells were harvested at 24 hpi, lysed, and analyzed by western blotting or qPCR analysis. **(C)** Analysis of the cells treated with heparin sodium salt. Cells were harvested at 24 hpi, lysed, and analyzed using western blotting or qPCR analysis. The number of viral gene copies in the sample volume was defined as per micrograms of total DNA. GAPDH was used as the endogenous reference. Values represent mean ± SD (*n* = 3) for experiments performed in triplicate. CCV, Channel catfish virus; qPCR, quantitative real-time PCR; GAPDH, glyceraldehyde-3-phosphate dehydrogenase; ORF39, CCV open reading frame 39. The p value for each study was determined by **P < 0.01 and ****P < 0.0001.

To determine whether the main adsorption factors are involved in CCV attachment, CCO/BB cells were incubated with hyaluronic acid, heparin sodium salt or collagen for 1 h and then infected with CCV for approximately 4 h, CCO/BB cells were harvested 24 h later and the amount of bound virus was detected using western blotting and qPCR analysis. As shown in Figure 2, the viral genome DNA copy number and protein expression levels were not significantly different after treatment with 0–500 μg/mL hyaluronic acid or collagen. The results suggested that hyaluronic acid and collagen are not associated with CCV attachment. heparin sodium salt reduced CCV adhesion at 5–10 mg/mL, and caused about 90% inhibition at 10mg/mL. The qPCR and western blot results showed the same trend. The copy number and protein expression levels were decreased, suggesting that heparan might play an important role in CCV attachment. To confirm that the exogenous adsorption factor plays an inhibitory role, different concentrations of collagen, hyaluronic acid, and heparin sodium salt were added after CCV infection for 4 h. Heparan exerted a competitive inhibitory effect on CCV adsorption to CCO/BB cells.

### Enzymatic digestion of cell surface heparan inhibits CCV genome replication and protein expression

**Figure 3:**
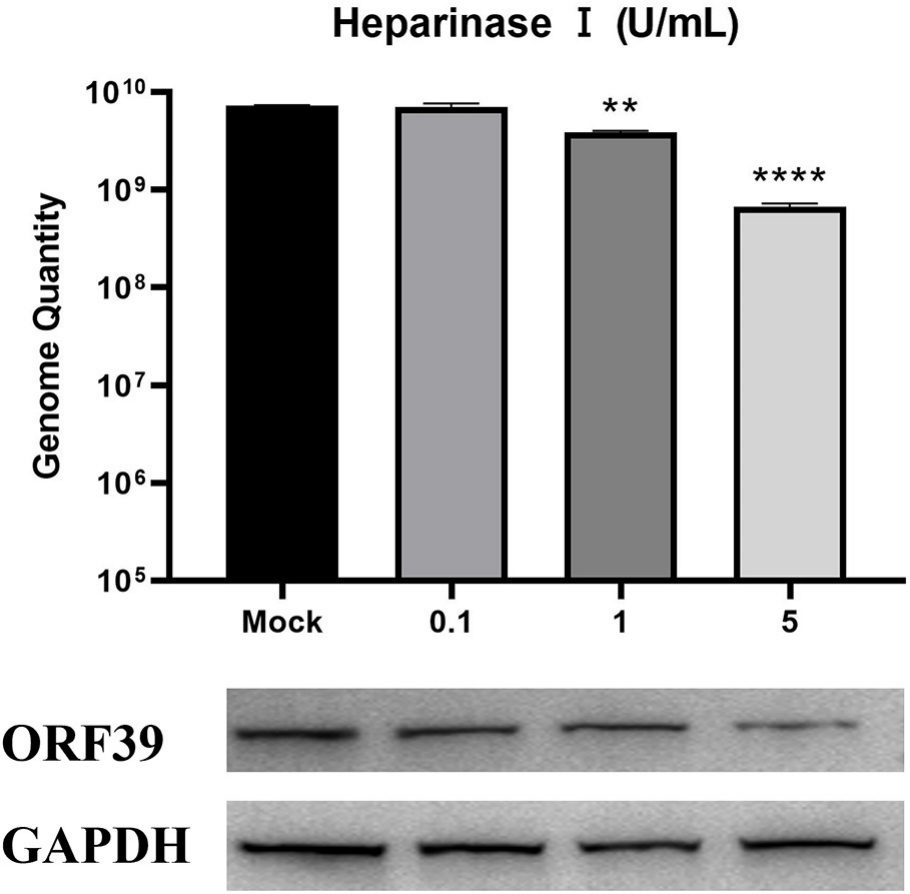
Enzymic digestion inhibits CCV attachment to CCO/BB cells. Channel catfish ovary cells were preincubated with heparinase I for 1 h. Analysis of the cells treated with heparinase I. Cells were harvested at 24 h post-infection (hpi), lysed, and analyzed by western blot or qPCR analysis. The number of viral gene copies in the sample volume was defined as per microgram of total DNA. GAPDH was used as the endogenous reference. Values represent mean ± SD (*n* = 3) for experiments performed in triplicate. CCV, Channel catfish virus; CCO/BB, channel catfish ovary cells; qPCR, quantitative real-time PCR; GAPDH, glyceraldehyde-3-phosphate dehydrogenase; ORF39, CCV open reading frame 39. The p value for each study was determined by **P < 0.01 and ****P < 0.0001.

Changes in ECM composition and structure can be mediated by proteases and have an impact on the overall structure and biomechanical properties of the formed network and cell signaling (28). If the interaction between heparin-like GAGs and the virus is required for viral infection, digestion of GAGs on the cell surface should inhibit infection. Cell surface heparan was removed using heparinase I, and CCV attachment was detected by western blotting and qPCR analysis. As shown in Figure 3, the viral gene copy number was significantly reduced in CCO/BB cells pretreated with heparinase I. At a heparinase concentration of 5 U/mL, the reduction was more than 90%, and the level of viral protein expression was also reduced. Thus, the ability of heparinase treatment to reduce viral gene copy numbers supports the notion that cell surface HS is a receptor for the virus. These results suggested that CCV attachment to the ECM is highly dependent on HS, which might be an important attachment factor for CCV infection.

### Heparan and heparinase effectively inhibit the production of offspring viruses

**Figure 4:**
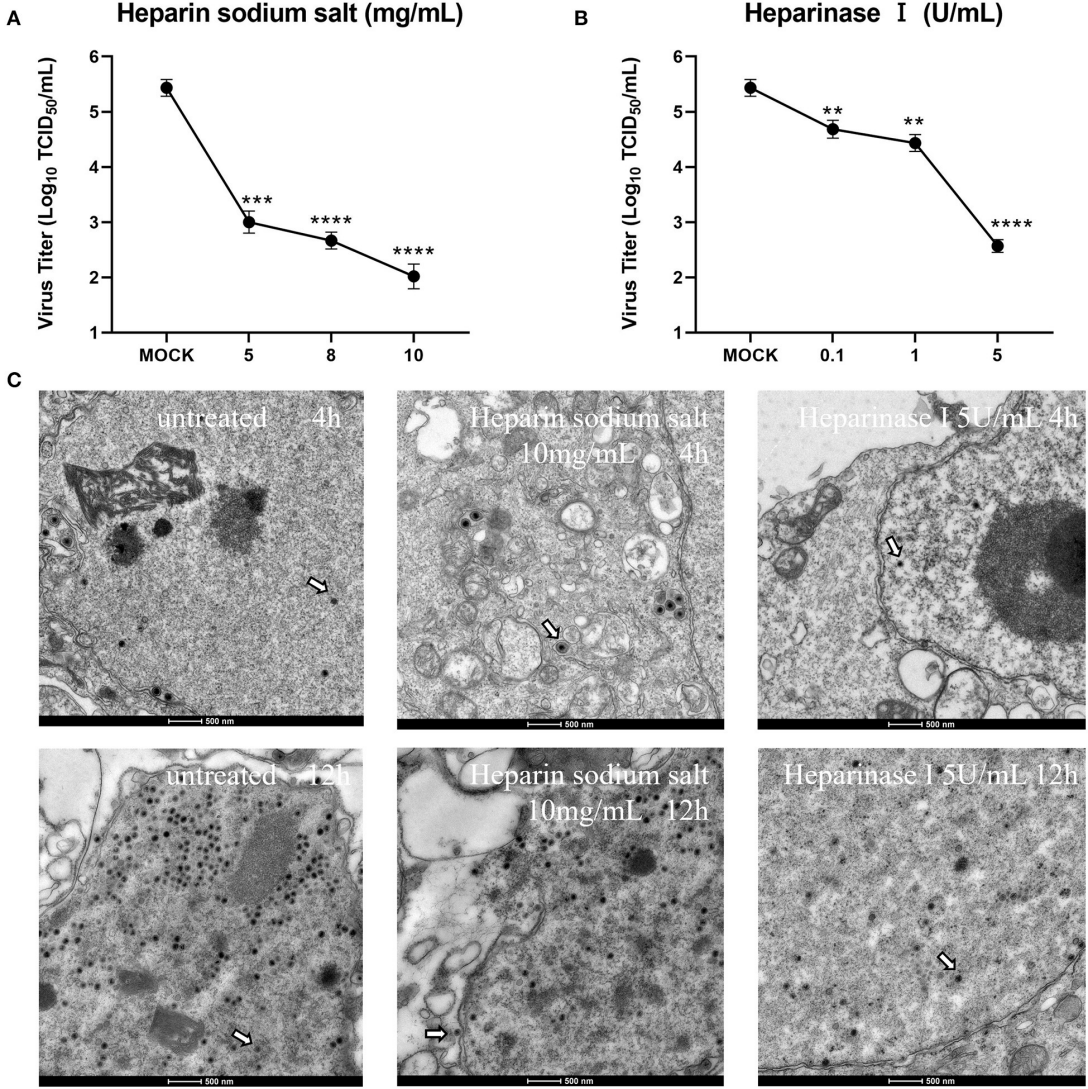
Effect of heparin sodium salt and heparinase I on CCV infection. **(A)** CCO/BB cells were pretreated with heparin sodium salt at 28°C for 1 h and then infected with CCV at 10-fold serial dilutions. Viral titers were determined using a plaque forming assay at 48 h post-infection (hpi). **(B)** CCO/BB cells were pretreated with heparinase I at 28°C for 1 h and infected with CCV at 10-fold serial dilutions. Viral titers were determined by plaque forming assay at 48 hpi. **(C)** The ultrastructure of CCV entering CCO/BB cells was observed using transmission electron microscopy. CCO/BB cells were pretreated with heparin sodium salt or heparinase I, or untreated, and then infected with CCV. The infected cells were observed and analyzed by transmission electron microscopy at 4 or 12 hpi. Arrows indicate CCV virions. Values represent mean ± SD (*n* = 3) for experiments performed in triplicate. CCV, Channel catfish virus; CCO/BB, channel catfish ovary cells; TCID_50_, median tissue culture infectious dose. The p value for each study was determined by **P < 0.01, ***P < 0.001, and ****P < 0.0001.

To further determine the effect of heparan on CCV infection of CCO/BB cells, viral particle release was measured after ~48 h of treatment using the TCID_50_ method. The CCV was serially diluted 10-fold, and the cells grown overnight in 96-well plates were incubated with different concentrations of heparin sodium salt or heparinase I for 1 h, the CCO/BB cells were then infected with different dilutions of CCV for about 4 h, and the culture medium was replaced. The cells were washed and treated for about 48 hours. The effect of heparin on CCV-infected CCO/BB cells was studied by observing the CPE produced by CCV-infected CCO/BB cells and calculating TCID_50_. The results showed that with the increase of heparin sodium salt or heparinase I concentration, the TCID_50_ value of CCV decreased significantly (Figures 4A, B), which further verified the important role of HS in CCV infection of CCO/BB cells. TEM images showed that the number of CCV particles in cells treated with 10 mg/mL heparin sodium salt and 5 U/mL heparinase I was lower than that in the control cells (Figure 4C and Supplementary Figure S1). Compared with the control group, the number of viruses in the treatment group decreased significantly after 4 h of infection. These results suggest that heparan and heparinase I can inhibit the production of offspring viruses.

### CCV virions can bind to heparan

**Figure 5:**
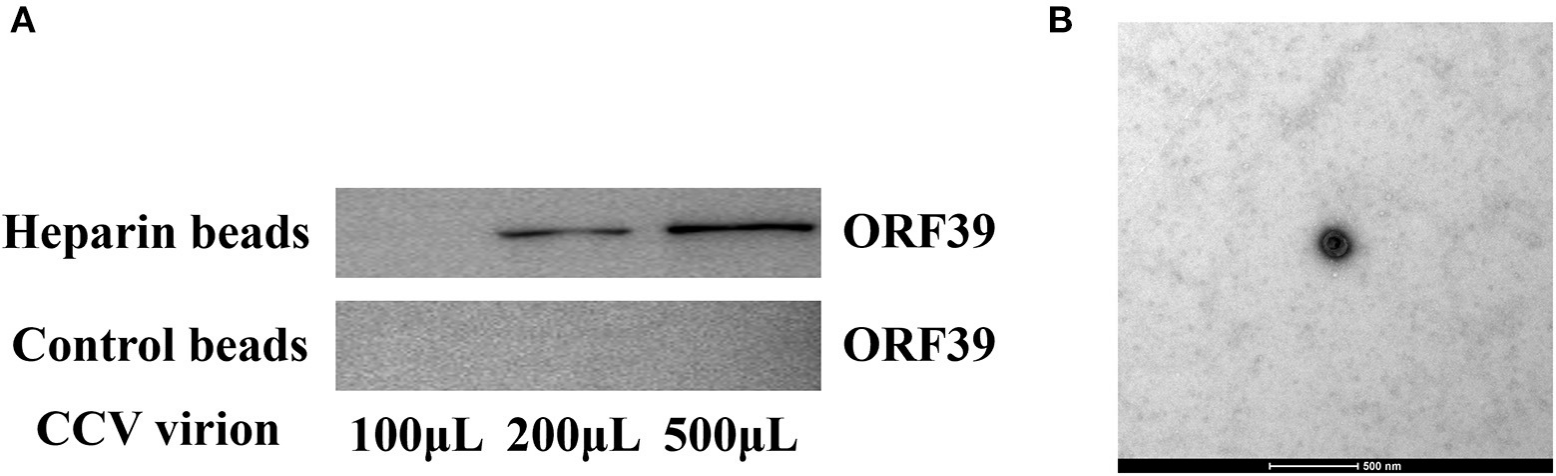
CCV virions bind heparin-Sepharose beads. **(A)** CCV virions are able to bind to heparan. Different doses of CCV virions were incubated with heparan-Sepharose beads for 1 h at 4°C. Unbound and eluted virions were collected and the amount of binding in the supernatant and eluate was determined using western blotting. **(B)** The ultrastructure of the virus particles was observed using transmission electron microscope. The virus solution eluted from the heparin-Sepharose beads 6FF was treated using the negative staining method, and the virus particles were observed and analyzed using transmission electron microscope. CCV, Channel catfish virus; ORF39, CCV open reading frame 39.

To further confirm that CCV can bind heparan into CCO/BB cells, heparan affinity chromatography was used to detect the interaction between CCV virions and heparin (29, 30). Western blotting results showed that CCV virions could bind well to heparan (Figure 5A), and with the increase in the number of CCV virions, the binding increased, while the control group had no obvious binding, indicating that CCV could bind to heparan in a dose-dependent manner, which was consistent with previous results. The virus solution eluted was negatively stained, and TEM showed that the virus particles were round or oval, with capsule and typical herpesvirus characteristics (Figure 5B).

## Discussion

Attachment is the first step in viral entry. Different viruses are able to use different ECMs to enter cells. The collection of extracellular molecules secreted by animal and plant cells is called the extracellular matrix (ECM). The ECM is usually composed of a well-organized network of polysaccharides and proteins that perform important functions in different tissues. It regulates adhesion and communication between cells. The ECM is the physical scaffold of cells, but it is also closely related to cellular physiological conditions, including homeostasis, survival, growth, migration, and differentiation (28, 31, 32). One of the major GAGs is heparan sulfate, a ubiquitous, highly negatively charged polysaccharide of the ECM that is involved in angiogenesis, embryonic development, and tissue repair (33, 34). HS binds to transmembrane or membrane-anchored proteins as proteoglycans to form heparan sulfate proteoglycans (HSPGs). Most viruses use HS as their primary attachment receptor (35, 36).Surface-exposed amino acid residues of human papillomavirus major capsid protein can mediate interaction between virions with cell surface HS (37). In 2009, Akhtar and Shukla discovered that the initial interaction of HSV is mediated through interaction with HS (38). In 2011, Hidari and Suzuki found that HS could enhance the entry efficiency of dengue virus (39). Many viruses, such as influenza viruses, reoviruses, adenoviruses, and polyomaviruses, use sialic acid-containing glycans as receptors for cell entry (40). Exogenous heparin molecules and analogs can regulate the affinity of HS and viral ligands, playing a certain role in controlling viral infection, and have potential antiviral value.

Enveloped viruses enter target cells by fusion of the viral and cellular membranes. Mainly divided into pH-dependent and independent mechanisms, pH-dependent conformational changes are often triggered by acidification within endosomes (41). Such conformational changes and membrane fusions are often caused by pH changes (42, 43). In previous studies (25), we found that CCV infects CCO/BB cells through a pH-dependent clathrin-mediated endocytosis pathway; however, whether this pH change could cause CCV to bind to some common adsorption factors for infection was unknown. In this study, gene copy number assays, enzymatic digestion experiments, and heparan affinity chromatography were used to investigate the role of major ECM components in CCV attachment. Our results showed that heparin sodium salt, a competitive inhibitor of HS, could significantly inhibit CCV attachment in a dose-dependent manner, whereas treatment of CCO/BB cells with hyaluronic acid and collagen had no significant effect on viral attachment. Using heparinase I to digest HS on the cell surface significantly prevent CCV adhesion, and the CCV virus titer also decreased with the increase of heparin sodium salt and heparinase I concentration. Meanwhile, the binding analysis of heparin-agarose beads and virions showed that CCV virions were able to bind heparin in a dose-dependent manner. These results suggest that HS may act as an attachment factor for CCV infection.

Attachment factors can trigger viral conformational changes and are critical for viral entry. The ECM plays a crucial role in regulating the active and reciprocal information exchange between cells (44). Our study showed that heparan is an important attachment factor associated with CCV infection of CCO/BB cells, and our work will increase our understanding of the mechanism of CCV attachment to CCO/BB cells and its interaction with receptors. There may be ubiquitously expressed molecules in cells that enable viruses to attach. The same virus might also choose different attachment factors in different cells and tissues. In this study, we selected three main attachment factors for research. An important role for HS was established; however, further screening is warranted. The specific attachment mechanism, sites, and interacting proteins of CCV infection of CCO/BB cells still require further study. Our research on CCV attachment and infection of CCO/BB cells could promote the development and use of antiviral drugs.

## Data availability statement

The datasets presented in this study can be found in online repositories. The names of the repository/repositories and accession number(s) can be found in the article/Supplementary material.

## Ethics statement

The manuscript presents research on animals that do not require ethical approval for their study.

## Author contributions

FY: Conceptualization, Funding acquisition, Supervision, Writing—review and editing. HC: Investigation, Methodology, Visualization, Writing—original draft. JX: Investigation, Methodology, Writing—review and editing. YW: Investigation, Writing—review and editing. CN: Investigation, Writing—review and editing. SS: Investigation, Writing—review and editing. LM: Investigation, Writing—review and editing. KH: Conceptualization, Investigation, Writing—review and editing. ZZ: Conceptualization, Funding acquisition, Project administration, Supervision, Writing—review and editing.
