# Supplementary material for: Heparan sulfate is the attachment factor associated with channel catfish virus infection on host cells
Source: Front Vet Sci. 2023 Sep 7;10:1260002. doi: 10.3389/fvets.2023.1260002 (PMC10514354; doi:10.3389/fvets.2023.1260002)
Supplement: Supplementary file 1 [file Data_Sheet_1.PDF]

Supplementary Material

1. Supplementary Figures

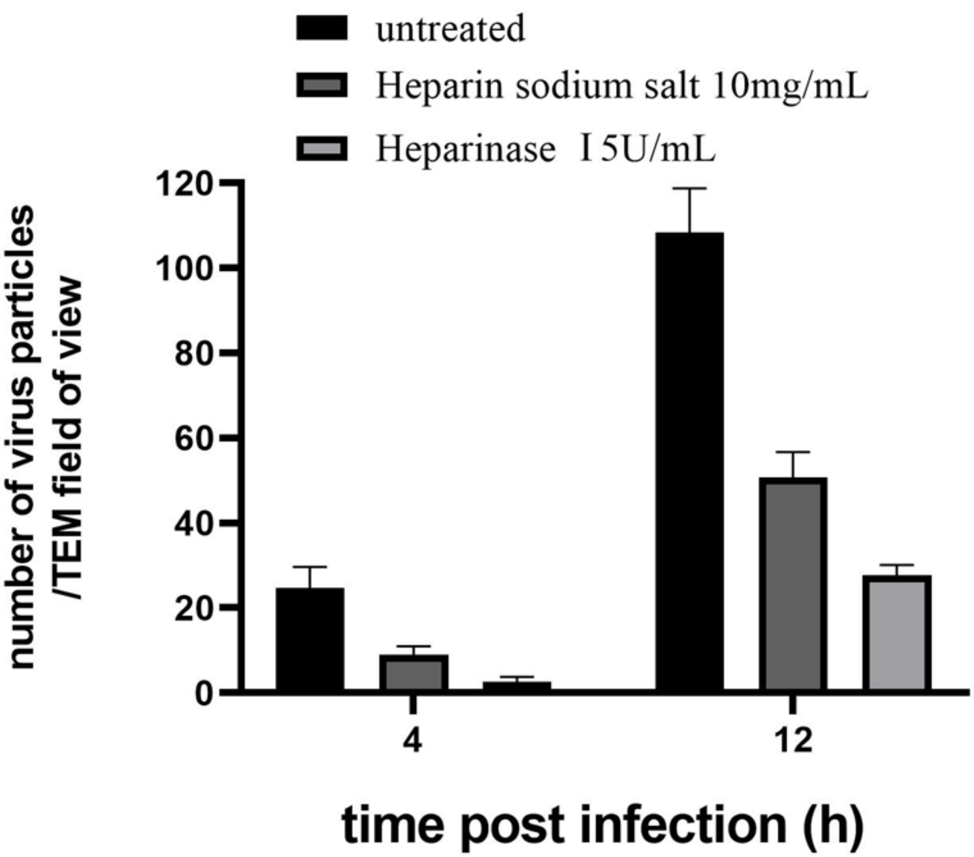

Supplementary Figure 1. Counts of virus particles in randomly selected fields of view in TEM assay (Related to figure 4). Values represent mean ± SD (n = 3) for experiments performed in triplicate.
